# The Effects of Algal Turf Sediments and Organic Loads on Feeding by Coral Reef Surgeonfishes

**DOI:** 10.1371/journal.pone.0169479

**Published:** 2017-01-03

**Authors:** Sterling B. Tebbett, Christopher H. R. Goatley, David R. Bellwood

**Affiliations:** College of Science and Engineering, and, ARC Centre of Excellence for Coral Reef Studies, James Cook University, Townsville, Queensland, Australia; Leibniz Center for Tropical Marine Ecology, GERMANY

## Abstract

Herbivorous and detritivorous fishes interact closely with the epilithic algal matrix (EAM) on coral reefs. While sediment and organic detrital loads within the EAM might influence this interaction, the responses of functionally distinct fishes to changing sediment and organic loads have not been investigated. Aquarium based feeding trials were performed to assess how different sediment and organic loads affected feeding by the highly abundant surgeonfishes, *Ctenochaetus striatus*, a detritivore, and *Acanthurus nigrofuscus*, a herbivore. *C*. *striatus* were highly sensitive to even small increases in sediment loads (of just 75 g m^-2^), displaying a significant decline in feeding rates as sediment loads increased. Although *C*. *striatus* is a specialised detritivore, changing organic loads had no effect and suggests that selection of feeding surfaces is primarily mediated by total sediment loads rather than organic loads. By contrast, *A*. *nigrofuscus* displayed no changes to its feeding behaviour regardless of sediment or organic load. These findings highlight the complex, species-specific way that sediments may mediate key ecological processes on coral reefs.

## Introduction

There is increasing recognition that fishes help maintain coral reef resilience, by performing an extensive range of important ecosystem functions [1,2]. Herbivory is regarded as a core component of these ecosystem functions as it controls the growth and expansion of algal communities [1,3,4]. Consequently, transitions from coral dominated to algal dominated reef systems are regularly attributed to reductions in rates of herbivory [3–6]. Overfishing of herbivorous fishes is often identified as the main driver leading to reduced rates of herbivory on reefs [1,2,7]. However, overfishing is just one of a number of factors which may lead to reductions in herbivory [8].

In some regions herbivorous fishes are not heavily targeted (e.g. the Great Barrier Reef [GBR]), however, declines in coral reef state and algal proliferation have still occurred [9–11]. Moreover, recent research has revealed that the epilithic algal matrix (EAM) on coral reefs may shift from a state of short productive algal turfs (SPATs) to a less desirable state of unpalatable long sediment-laden algal turfs (LSATs), without any change in the abundance of herbivorous fishes [8]. Although subtle, this transition to LSATs may alter fundamental reef processes including coral recruitment [12–14] and benthic interactions [15,16], reducing the resilience of reefs to other environmental perturbations. The main driver behind this shift appears to be an increase in benthic sediment loads within the EAM [8].

Although sediments occur naturally on all coral reefs, changing land use practices and increased coastal development have led to increased sediment inputs to coral reefs on the GBR [17–19]. Sediment inputs from terrestrial runoff have increased by more than 500% since European settlement in the GBR catchment area [19,20]. Moreover, in the decade preceding 2014, 25 million cubic metres of dredge spoil was dumped within the GBR World Heritage Area [17]. How these increased sediment inputs relate to the sediment loads trapped within EAMs is currently unclear. However, recent evidence has documented a 3700% increase in EAM sediment loads on an inner-shelf reef of the GBR during the last decade [8]. Clearly there is a need to further understand how sediments affect important benthic processes and interactions on coral reefs.

Sediments within the EAM have long been considered an important factor which may influence feeding rates of herbivorous/detritivorous fishes [21], yet we know surprisingly little about the nature of this interaction. A clear positive relationship exists between sediment load and algal turf length on reefs [8,22–24], and sediments may facilitate algal growth by mediating herbivory rates [8,23,25]. Reductions of EAM sediment loads on coral reefs result in significantly increased herbivorous/detritivorous fish feeding rates [26,27]. However, how sensitive herbivorous/detritivorous fishes are to sediment loads, and the reasons behind this sensitivity, remain unclear. Furthermore, in addition to inorganic sediments, the EAM also contains organic detritus [28], a food resource with a high nutritional value [29–31], which may also play an important role in the relationship between the EAM and the feeding rates of fishes. Unsurprisingly, some fish species specifically target this resource and primarily ingest organic detritus [32,33]. Research suggests that the proportion of benthic particulates (sediment and detritus) composed of organic detritus could be a critical factor that influences feeding rates by fishes, as increasing inorganic sediment loads can dilute detrital loads, consequently decreasing the net nutritional value of the EAM [29,34]. However, our knowledge of these interactions is currently limited to paired feeding choice trials on the surf parrotfish, *Scarus rivulatus*, a common herbivorous/detritivorous fish on the inner-shelf of the GBR [34]. The relationship between organic load and feeding rate in other species is unclear. In particular, we lack information on how organic loads interact with sediment loads and how this affects fish species that are common on mid- and outer-shelf reefs.

**Fig 1:**
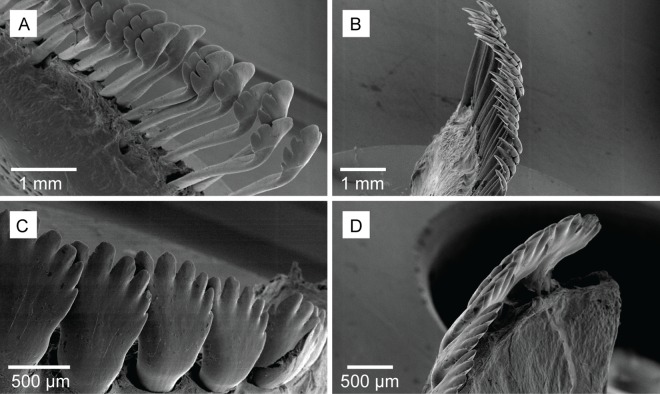
Scanning electron micrographs of *Ctenochaetus striatus* and *Acanthurus nigrofuscus* teeth. *C*. *striatus* teeth (A) anterior view of dentary, (B) lateral view of dentary and *A*. *nigrofuscus* teeth (C) anterior view of dentary and (D) lateral view of dentary.

Many herbivorous and detritivorous fishes show distinct distribution patterns across environmental gradients [35–37]. On the GBR relatively few species are common on inner-shelf reefs, and both diversity and density of herbivorous fishes is higher on the mid- and outer-shelf reefs [38–40]. Surgeonfishes (Acanthuridae) display this typical pattern, being uncommon or absent from inner-shelf reefs, while they are often the most abundant herbivorous/detritivorous fishes on mid- and outer-shelf reefs [39,41,42]. Here, surgeonfish species feed on the EAM with specialised morphologies and/or behaviour allowing them to target different food resources [43–45]. This diversity of feeding modes is exemplified by the superficially similar, *Ctenochaetus striatus* and *Acanthurus nigrofuscus*; two abundant surgeonfishes with widespread distributions across the Indo-Pacific [46,47]. *A*. *nigrofuscus* is herbivorous, using short nipping bites and spatulate teeth (Fig 1C and 1D) to remove algal matter from EAMs, while *C*. *striatus* is a specialised detritivore using elongate, comb-like teeth (Fig 1A and 1B) to brush detritus from the EAM, leaving algal turfs intact [43,48]. Previous research suggests that surgeonfishes may be highly sensitive to EAM sediment loads [27]. While this suggests sensitivity as a group, we currently do not know how different members of the family respond to increased sediment loads. The aim of this study, therefore, is to examine the effects of increasing sediment loads and their interaction with organic loads on two functionally distinct surgeonfish species; the detritivore *C*. *striatus* and the herbivore *A*. *nigrofuscus*.

## Materials and Methods

### Ethics statement

The current study was conducted in accordance with the animal ethics guidelines of James Cook University, Townsville, including authorisation to collect, observe, maintain in captivity, feed and film the study organisms under the animal ethics approval number A2181, and the permitting requirements of the Great Barrier Reef Marine Park Authority (permit number: G12/35293.1). In order to minimise discomfort and ensure optimal condition of the study organisms, they were maintained using routine monitoring, feeding and cleaning protocols in accordance with the authorised ethical procedures. Data are available in S1 Table.

### Experimental procedures

#### Fish collection and husbandry

Feeding experiments were performed on *C*. *striatus* and *A*. *nigrofuscus* at Lizard Island Research Station (14° 40′ S, 145° 27′ E) on the mid-shelf of the GBR. 20 *C*. *striatus* and 14 *A*. *nigrofuscus* were caught using a barrier net. The average total length of *C*. *striatus* and *A*. *nigrofuscus* used in the feeding trials was 112.8 ± 6 mm (± SE) and 130.4 ± 2 mm, respectively. The fishes were held at Lizard Island Research Station in individual 90 L containers (600 × 400 × 380 mm) with flow-through water. Fishes were provided with natural EAM-covered surfaces on which to graze and also fed a frozen marine fish food designed for herbivorous/detritivorous fishes. Once fishes were feeding repeatedly on EAM-covered surfaces (usually within two days) trials were started.

#### Treatment preparation

Benthic particulate treatments were prepared following [34]. To ensure similar properties to sediments found naturally in Lizard Island EAMs, benthic sediment was collected from Lizard Island lagoon in 2–4 m of water. To remove residual organic matter, sediments were bleached using hydrogen peroxide (H_2_O_2_) (which spontaneously decomposes into water and oxygen minimising the potential for residual odour), for more than two weeks. Sediment was then dried to a constant weight at 60°C and sieved through a sieve stack (2000–63 μm).

Hikari Marine A (a commercially available marine fish food pellet) was used to produce organic material which approximates the nutritional composition of EAM detrital aggregates [31,49] and acts as a substitute for detritus in reef particulates [34]. Hikari Marine A was ground using a pestle and mortar and then passed through a 125 μm sieve to ensure similar particle sizes to natural detrital material [31,34]. Using the prepared sediment and organic material, benthic particulate loads were created by weighing out individual grain size fractions to simulate grain size distributions for Lizard Island reef crest EAM sediments [22] with all grain sizes under 2000 μm considered sediment (sands, silts and clays; ISO 14688–1:200). Sediment grain size distributions from the reef crest were used, as this is the preferred feeding habitat of the two focal fish species [35,41,50].

To examine how sediment loads influenced the feeding responses of *C*. *striatus* and *A*. *nigrofuscus*, feeding trials were performed using twelve benthic particulate treatments comprising six different sediment loads (75–450 g m^-2^, in 75 g m^-2^ increments) at two different organic loads (2% and 14%). These sediment load treatments corresponded to natural levels: 75 g m^-2^ is the lowest EAM sediment load published to date from mid-shelf reefs [50], while 450 g m^-2^ represents an upper load, and is approximately half the sediment load found in inner-shelf reef crest EAMs [8,22,27,50]. Organic loads of 2% and 14% represent the lower and upper values of organic detritus recorded from benthic particulates from Lizard Island EAMs [29].

As organic loads have been found to affect the feeding rates of other detritivorous fishes [34], we also assessed in more detail the feeding response of *C*. *striatus*, a specialised detritivore, to changing organic loads. This was achieved using feeding trials with six different organic loads (2–22%, in 4% increments) at a fixed sediment load of 225 g m^-2^ (which approximates the average sediment load in Lizard Island reef crest EAMs [27]). The organic loads represented natural levels and although 14% represents the upper percentage of organic detritus reported from Lizard Island EAMs [29], higher organic loads have been found on some reefs of the GBR [23,30]. Therefore we also used organic loads of 18 and 22%, which could potentially be found in EAMs on mid-shelf reefs.

#### Experimental process

A repeated measures design was used, where each replicate fish was exposed to all of the treatment levels, in either the sediment load (12 individual treatments, i.e. 6 sediment loads at 2 different organic loads) or organic load experiments (6 individual treatments). Replicate fishes were exposed to each of the individual treatments in a given experiment once and were only exposed to one treatment per day. Treatments were fully randomised between the replicate fish and days. Following each day’s experimental treatment the fish were fed to satiation using a frozen marine fish food designed for herbivorous/detritivorous fishes to equilibrate their diets following different treatments. Aquaria were then syphoned clean to remove uneaten food and waste material. For *C*. *striatus*, twelve replicate fishes were used for sediment load experiments and eight replicate fishes for organic load experiments, while for *A*. *nigrofuscus* fourteen replicate fishes were used for sediment load experiments.

Benthic particulate treatments were offered to fish on pre-conditioned natural feeding surfaces (circular 50 cm^2^, flat, EAM-covered coral rocks, free of large macroalgae or encrusting organisms). Prior to use, all rocks were rinsed in seawater to remove any sediment and detritus from the EAM and subsequently were visually inspected to ensure that they were well covered with turfing algae. Feeding rocks were then randomly allocated to individual tanks. One rock was placed at the end of each aquarium and concealed within a 90 mm diameter polyvinyl chloride (PVC) pipe to prevent feeding by fishes. The pre-prepared benthic particulates (sediment and organic particulates) were wetted and then poured into the PVC pipe and allowed to settle for at least 17 hours. At the same time each day (1100–1200 hours), a video camera (GoPro) was placed into each aquarium, the PVC pipe was removed and a 10 mm high PVC ring was placed over the feeding rock to restrict feeding to the upper surface. The methods follow [34]. Feeding behaviour was recorded for a minimum of two hours.

### Video analysis

To account for differences in acclimation times among individual fishes following the exposure of feeding surfaces, video footage was examined for fifteen minutes from the first bite taken by the fish on the rock (which usually took less than one minute). Three independent metrics were assessed. The first, ‘bite rate’, measured the feeding rate of the fishes, i.e. bites from the surface of the rock per minute. The second, ‘bites rejected’, assessed the number of bites that fish took and then subsequently rejected. Rejected bites were quantified by recording the number of sediment spits (the forceful rejection of sediment from the mouth *sensu* [51]). Rejected bites were standardised to percent of total bites, to account for differences in total feeding rates on each rock. The third, ‘multiple bites’, assessed if fish were discouraged from feeding on a particular treatment after a single exploratory bite. The number of feeding bouts with a single bite was compared to the number of feeding bouts with multiple consecutive bites.

### Statistical analyses

Generalised linear mixed effects models (GLMMs) were used to analyse sediment and organic load data. First, bite rate data were examined in response to sediment or organic loads using GLMMs with negative binomial error distributions, accounting for the non-normal distribution and overdispersion of the bite data. Data on the percentage of bites rejected ‘sediment spits’ and the percentage of feeding bouts with multiple bites were analysed using GLMMs with a binomial error structure. Where overdispersion was detected during the modelling process an observation level random effect was employed in binomial models [52].

For models examining response to sediment load, full models were initially fitted. Sediment load was treated as a fixed continuous factor (following [53]), organic load as a fixed categorical factor (two levels) and individual fish as a random factor (to account for the repeated measures design of the study and subsequent non-independence), with an interaction between fixed factors. In all cases, models were simplified based on the corrected Akaike Information Criterion (AICc). For models examining response to organic load, organic percentage was treated as a fixed continuous factor (following [53]) and individual fish as a random factor. Model fits were evaluated using residual plots. All statistical analyses were performed in the software R [54], using the *lme4* [55], *nlme* [56] and *AICcmodavg* [57] packages.

## Results

### Ctenochaetus striatus

#### Sediment load experiment

**Fig 2:**
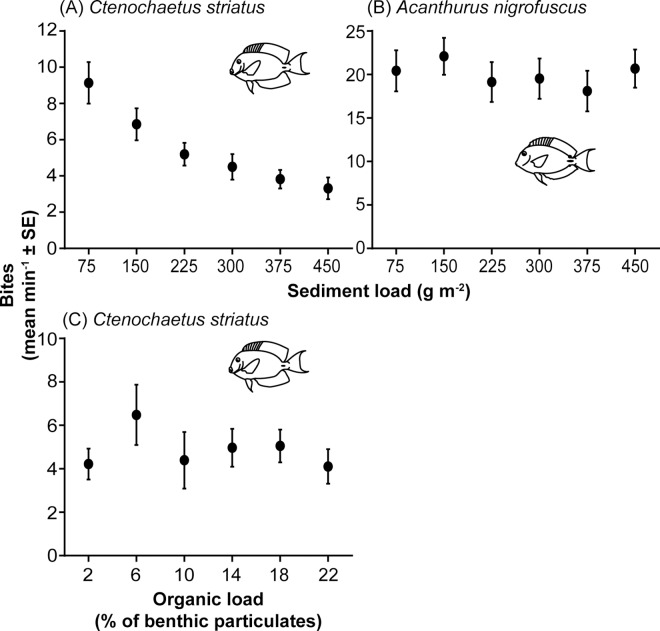
The effects of algal turf sediment and organic loads on feeding rates of surgeonfishes. Bite rate (mean bites min^-1^ ± SE) of (A) *Ctenochaetus striatus* (n = 24) and (B) *Acanthurus nigrofuscus* (n = 28) on six different sediment loads and (C) *C*. *striatus* (n = 8) on six different organic loads.

*C*. *striatus* were highly sensitive to increasing sediment loads within the EAM (GLMM; *t* = -6.35, *p* < 0.0001; S2 Table). A total of 11,817 bites were recorded from *C*. *striatus* and revealed that the bite rate declined in an exponential manner from 9.1 ± 1.1 bites min^-1^ (mean ± SE) on rocks with sediment loads of 75 g m^-2^ to 3.3 ± 0.6 bites on rocks with sediment loads of 450 g m^-2^ (Fig 2A). Based on the AICc scores the final model contained only sediment load as a factor, suggesting neither the interaction between organic load and sediment, nor organic load by itself had a significant effect (S3 Table).

**Fig 3:**
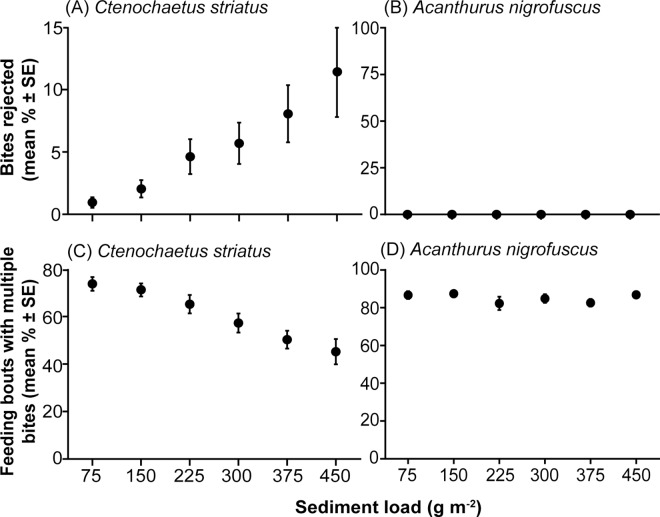
The behavioural response of two surgeonfishes to increasing sediment loads. The percentage of bites rejected (mean ± SE) by (A) *Ctenochaetus striatus*, (n = 24) and (B) *Acanthurus nigrofuscus*, (n = 28) and the percentage of feeding bouts with multiple bites (mean ± SE) by (C) *C*. *striatus* (n = 24) and (D) *A*. *nigrofuscus* (n = 28).

*C*. *striatus* increasingly rejected bites as sediment load increased, with the percentage of bites resulting in sediment rejection increasing from 1.0 ± 0.4% on rocks with sediment loads of 75 g m^-2^ to 11.4 ± 3.6% on rocks with a sediment load of 450 g m^-2^ (Fig 3A). Again, based on the AICc the final model contained only sediment load as a factor (GLMM; *z* = 7.27, *p* < 0.0001; S2 Table and S3 Table).

With increasing sediment load *C*. *striatus* also exhibited fewer feeding bouts with multiple bites. The percentage of bouts with multiple bites decreased from 73.8 ± 2.9% on rocks with sediment loads of 75 g m^-2^ to 45.2 ± 5.3%, on rocks with sediment loads of 450 g m^-2^ (Fig 3C). Again, based on the AICc the final model contained only sediment load as a factor (GLMM; *z* = -6.92, *p* < 0.0001; S2 Table and S3 Table).

#### Organic load experiment

Varying the organic load had no discernible effects on the feeding behaviour of *C*. *striatus*. For the bite rate (Fig 2C), percentage of bites resulting in sediment spitting, and percentage of feeding bouts with multiple bites, no models were statistically significant (S2 Table). Furthermore, the models used to examine bite rate and feeding bouts with multiple bites performed no better than the null model, based on the AICc, suggesting that organic percentage had little effect on feeding in *C*. *striatus* (S3 Table).

#### Acanthurus nigrofuscus

A total of 50,376 bites were recorded from *A*. *nigrofuscus*. Varying sediment and organic loads within the EAM had no detectable effects on its feeding behaviour. No models were statistically significant (S4 Table) and based on the AICc no models performed better than the null model in terms of bite rate (Fig 2B; S5 Table), or in terms of feeding bouts with multiple bites (Fig 3D, S5 Table). Sediment rejection (spitting) was never observed in any of the treatments (Fig 3B). These results suggest that sediment and organic loads had little effect on feeding by *A*. *nigrofuscus*.

## Discussion

While *C*. *striatus* were highly sensitive to increasing sediment loads within the EAM, *A*. *nigrofuscus* were unaffected. Neither species displayed strong sensitivity to variation in organic loads, which contrasts markedly to previous studies using the parrotfish *S*. *rivulatus* [34]. These results highlight how EAM sediment loads affect two superficially similar species in fundamentally different ways, providing a more nuanced understanding of how fish interact with EAMs on coral reefs.

### Response of *Ctenochaetus striatus*

The effects of increasing EAM sediment loads on the feeding behaviour of *C*. *striatus* were consistent with previous ecological evidence [26,27,50]. Across the continental shelf, and among habitats, *C*. *striatus* display distinct distributional patterns. They predominantly inhabit the reef crest of mid- and outer-shelf reefs on the GBR [35,39,42], where EAM sediment loads are lowest [22,27]. *C*. *striatus* are rarely found on inner-shelf reefs [35,39], where EAM sediment loads are highest [8,58]. The sensitivity to sediments displayed by *C*. *striatus* under experimental conditions strongly suggests that EAM sediment loads play a significant role in shaping the distribution of this species. While suspended sediments might contribute to the distribution of fish species [42,59,60] as they effect the settlement cues [61,62] and development of larval fishes [63,64], our data suggest that once a fish settles, EAM sediment loads can also determine the potential availability of feeding surfaces. High sediment loads may suppress feeding, limiting a fish’s ability to procure nutritional resources.

**Fig 4:**
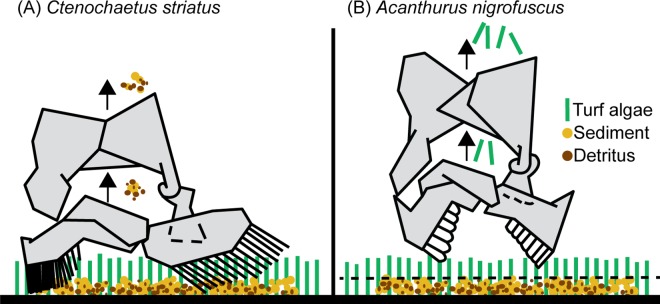
A schematic diagram showing how different surgeonfishes interact with sediments in algal turfs. (A) *Ctenochaetus striatus* ingests sediments as it brushes detritus from algal turfs, while (B) *Acanthurus nigrofuscus* crop algal turfs above sediments. Diagrams of the jaw schematics redrawn from [48].

On coral reefs, *C*. *striatus* are likely to be more responsive to EAM sediments than most other herbivorous/detritivorous fishes because of their unusual feeding mode [48,51]. The manner in which *C*. *striatus* feed, by brushing detritus from the EAM using specialised comb-like teeth, means that they directly remove and ingest the fine particulate material contained within the EAM [43,48,65] (Fig 4A). The mechanism by which sediments suppress feeding by *C*. *striatus* does not, however, appear to be driven by the dilution of organic matter, as seen in the parrotfish, *S*. *rivulatus* on inner-shelf reefs [34]. Surprisingly, *C*. *striatus* appeared to be largely unaffected by changes to organic loads. The present study used the same methods and the same organic matter as [34], consequently the contrasting results are unlikely to be due to methodological differences. The results of the current study may instead reflect the ability of one species to tolerate sediment loads where another species cannot.

The surf parrotfish *S*. *rivulatus* are predominantly found on inner-shelf reefs [38,39] where EAM sediment loads are far higher than on mid-shelf reefs [8,58]. Consequently they are likely to have behavioural, morphological and/or physiological modifications that enable them to deal with high sediment loads. The main factor that drives feeding selectively in *S*. *rivulatus* appears to be in locating EAMs with high proportions of organic material, which may be the key to survival where high sediment loads are common [34]. In contrast, *C*. *striatus* are found in reef habitats with some of the lowest sediment loads. It appears that the factor that predominantly deters feeding by *C*. *striatus* is total sediment load, not available organics. The reason behind the sensitivity of *C*. *striatus* to total sediment loads is unclear, but it is likely to be related to the unusual manner in which these fishes feed and process ingested material. *C*. *striatus* are specialised at removing fine particulates from the EAM [43,48,65] and it could be that sediment impairs their ability to adequately procure nutritional resources [48,65,66] or that processing high loads of sediments is energetically costly [21].

### Response of *Acanthurus nigrofuscus*

In contrast to *C*. *striatus*, *A*. *nigrofuscus* were unaffected by the sediment loads used in the present study. This may explain the broader habitat associations of *A*. *nigrofuscus* which can be found in high densities on reef habitats other than the crest, including the reef flat and back-reef habitats of mid- and outer-shelf reefs of the GBR [35,39,42]. In these habitats, EAM sediment loads are far higher than on the reef crest [22,27]. The ability of *A*. *nigrofuscus* to live in areas with higher sediment loads may be explained by the way it interacts with the EAM.

Unlike *C*. *striatus*, *A*. *nigrofuscus* are able to avoid the sediment contained within the EAM as they use short, quick bites to nip off algal strands protruding from the surface of the EAM [32,48,51]. This inference is supported by dietary and behavioural evidence which shows that this species ingests little sediment when feeding [32] and exhibits no sediment rejection behaviour (Fig 3B). This manner of interacting with the EAM would suggest that, as long as algal strands protrude from the EAM, *A*. *nigrofuscus* can readily access them and will be unaffected by the underlying sediment load and composition (Fig 4B). On coral reefs, a strong, positive relationship exists between algal turf length, and EAM sediment load [22–24] and it is suggested that EAM sediments control algal turf length by suppressing herbivory [8,24]. However, this relationship varies depending on the herbivorous fish involved. Cropping grazers such as *A*. *nigrofuscus*, which nip off algal strands above the sediment, may control algal length regardless of sediment depth. However, they only control algal length protruding above the sediment. By contrast, parrotfishes remove and ingest the entire EAM (sediments, turf algae and detritus) [67] but will only feed where there is a high proportion of organic material in the sediment [8,34].

### Potential ecological implications

Sediment inputs to coral reef systems are increasing in many regions around the world, including the GBR [18,20,68]. These sediments arise because of human activities such as dredging and coastal development as well as activities that modify terrestrial runoff such as land clearing and intensive farming practices [17–20,69]. If these sediments accumulate within the EAM on reefs they may have implications for the resilience of these environments by altering interactions between fishes and the EAM. It has been suggested that EAM sediments decrease the resilience of coral reefs by supressing feeding by herbivorous fishes [26,27]. While this explanation may be true for some species, the results of the present study suggest that it does not apply to all herbivorous/detritivorous fishes to the same extent. Rather than asking how much sediment suppresses herbivory on EAMs, in some cases it may be more ecologically relevant to ask; do increased sediment loads limit the ability of fishes to perform their functional role (e.g. crop algal turfs)? Evidently, the way increased sediment loads affect fishes hinges on the functional capabilities of the fishes in question. We now have preliminary information on how sediments affect representative herbivorous/detritivorous fishes which brush, crop and scrape the EAM.

It appears that for fishes which selectively brush detrital aggregates from the EAM (e.g. *C*. *striatus*), high sediment loads reduce their feeding activity. For fishes which crop algal turfs, e.g. *A*. *nigrofuscus*, their ability to crop algal strands which protrude from the sediments means they are unaffected by total sediment or organic load. Finally, feeding by scraping species such as *S*. *rivulatus* is affected by the nutritional quality of the EAM [8,34]. It must be noted that further research is necessary to evaluate the extent to which these findings apply to other functionally similar species, however the representative species studied so far are among the most abundant herbivorous/detritivorous fishes on the GBR [35,39].

As sediment affects functionally disparate herbivorous/detritivorous fishes differently, increases in EAM sediment loads on reefs may alter a number of functional processes. In brushing detrital aggregates from the EAM, *C*. *striatus* removes, ingests and exports large quantities of EAM bound sediment off the reef [50,70]. However, if sediment loads become too high, this process may cease [8,50]. Although increased sediment loads may not manifest themselves as a reduction of feeding rates in cropping herbivorous fishes, high sediment loads in the EAM may prevent these fishes from effectively removing algae, consequently making it harder to maintain short algal turfs on coral reefs [8,71]. Compared to croppers, scrapers remove the entire EAM leaving behind bare reef matrix, slowing the development of deep algal communities and allowing the settlement of other coral reef organisms [72–74]. If sediment loads dilute the nutritional value of the EAM, this function may be lost [34]. By impeding a variety of functional roles, sediments can disrupt ecosystem processes and drive a transition to LSATs on coral reefs which are largely avoided by fishes and may represent an alternative stable state [8,26]. Evidence suggests that transitions of the EAM to LSATs may occur without any changes to the abundance and community composition of the fishes which maintain the EAM [8] and therefore managing functionally important fishes against overfishing may not be adequate to ensure resilient reefs. Management of other stressors such as sediment inputs is also necessary to ensure reef resilience.

As sediment inputs onto coral reefs continue to increase globally, we are likely to observe a range of ecological impacts. By mediating the functional roles which fishes fulfil on reefs, increasing sediment loads are likely to substantially impair their resilience. Understanding the differences in the interactions to sediments and algae among fish species is critical in revealing how increased sediment inputs can impact ecological processes on coral reefs and consequently how this stressor can reduce reef resilience in the face of global climate change.

## Supporting Information

S1 Table Raw data. Sediment load and organic percentage indicate the specific benthic particulate treatment the fish was exposed to. All count data is the total number recorded during the 15 minute recording period.
(PDF)

S2 Table Summary of GLMM results used to examine the effects of sediment and organic loads on *Ctenochaetus striatus*. SE = standard error. Negative binomial models used a *t* statistic while binomial models used a *z* statistic.
(PDF)

S3 Table Comparison of GLMMs used to examine the response of *Ctenochaetus striatus* to sediment and organic loads. Models are compared using the corrected Akaike Information Criterion (AICc). Shown are degrees of freedom (df), model maximum log-likelihood (logLik), AICc, change in AICc (Δ) and AICc weight (wAICc).
(PDF)

S4 Table Summary of GLMM results used to examine the effects of sediment and organic loads on *Acanthurus nigrofuscus*. SE = standard error. Negative binomial models used a *t* statistic while binomial models used a *z* statistic.
(PDF)

S5 Table Comparison of GLMMs used to examine the response of *Acanthurus nigrofuscus* to sediment and organic loads. Models are compared using the corrected Akaike Information Criterion (AICc). Shown are degrees of freedom (df), model maximum log-likelihood (logLik), AICc, change in AICc (Δ) and AICc weight (wAICc).
(PDF)
