# Supplementary material for: The Effects of Algal Turf Sediments and Organic Loads on Feeding by Coral Reef Surgeonfishes
Source: PLoS One. 2017 Jan 3;12(1):e0169479. doi: 10.1371/journal.pone.0169479 (PMC5207718; doi:10.1371/journal.pone.0169479)
Supplement: S1 Table — Sediment load and organic percentage indicate the specific benthic particulate treatment the fish was exposed to. All count data is the total number recorded during the 15 minute recording period. (PDF) [file pone.0169479.s001.pdf]

S1 Table. Raw data. Individual treatments are denoted by sediment load (g m^{-2}) and organic (%). The counts for total bites, number of bouts and spits are the total recorded in the 15 minute recording period.

Fish Species	Fish ID	Sediment Load (g m^{-2})	Organic (%)	Total Bites	Number of			
					Bouts	Bouts With >1 bites	Bouts With <1 bite	Spits
C. striatus	1	75	2%	240	53	35	18	0
C. striatus	1	150	2%	68	29	17	12	4
C. striatus	1	225	2%	33	11	9	2	1
C. striatus	1	300	2%	148	52	44	8	3
C. striatus	1	375	2%	53	32	12	20	4
C. striatus	1	450	2%	73	42	16	26	5
C. striatus	2	75	2%	246	33	31	2	0
C. striatus	2	150	2%	119	24	20	4	0
C. striatus	2	225	2%	99	22	19	3	3
C. striatus	2	300	2%	52	15	11	4	0
C. striatus	2	375	2%	74	34	18	16	2
C. striatus	2	450	2%	107	38	25	13	4
C. striatus	3	75	2%	39	11	8	3	0
C. striatus	3	150	2%	126	37	27	10	0
C. striatus	3	225	2%	47	12	9	3	1
C. striatus	3	300	2%	31	18	9	9	1
C. striatus	3	375	2%	26	10	7	3	1
C. striatus	3	450	2%	51	30	11	19	0
C. striatus	4	75	2%	120	35	24	11	3
C. striatus	4	150	2%	23	11	6	5	2
C. striatus	4	225	2%	79	32	14	18	6
C. striatus	4	300	2%	178	34	31	3	1
C. striatus	4	375	2%	72	30	15	15	7
C. striatus	4	450	2%	60	29	16	13	8
C. striatus	5	75	2%	172	39	33	6	2
C. striatus	5	150	2%	77	16	12	4	0
C. striatus	5	225	2%	113	33	27	6	3
C. striatus	5	300	2%	67	22	13	9	4
C. striatus	5	375	2%	51	26	12	14	2
C. striatus	5	450	2%	25	14	6	8	1
C. striatus	6	75	2%	52	14	10	4	0
C. striatus	6	150	2%	88	23	16	7	1
C. striatus	6	225	2%	58	25	14	11	4
C. striatus	6	300	2%	18	9	2	7	1
C. striatus	6	375	2%	100	25	21	4	2
C. striatus	6	450	2%	26	8	8	0	0
C. striatus	7	75	2%	211	41	31	10	1
C. striatus	7	150	2%	100	30	19	11	4

C. striatus	7	225	2%	83	31	17	14	0
C. striatus	7	300	2%	83	32	18	14	4
C. striatus	7	375	2%	71	34	16	18	14
C. striatus	7	450	2%	27	17	6	11	5
C. striatus	8	75	2%	113	29	21	8	0
C. striatus	8	150	2%	108	39	27	12	2
C. striatus	8	225	2%	144	41	31	10	0
C. striatus	8	300	2%	26	14	4	10	3
C. striatus	8	375	2%	89	44	19	25	4
C. striatus	8	450	2%	10	7	3	4	0
C. striatus	9	75	2%	155	38	21	17	4
C. striatus	9	150	2%	63	20	14	6	0
C. striatus	9	225	2%	45	18	13	5	2
C. striatus	9	300	2%	34	18	8	10	0
C. striatus	9	375	2%	149	48	33	15	1
C. striatus	9	450	2%	19	12	3	9	4
C. striatus	10	75	2%	122	21	16	5	0
C. striatus	10	150	2%	50	19	14	5	0
C. striatus	10	225	2%	25	14	7	7	1
C. striatus	10	300	2%	49	27	13	14	8
C. striatus	10	375	2%	43	18	9	9	0
C. striatus	10	450	2%	20	14	3	11	3
C. striatus	11	75	2%	103	28	21	7	1
C. striatus	11	150	2%	85	26	19	7	4
C. striatus	11	225	2%	99	21	18	3	2
C. striatus	11	300	2%	52	21	15	6	11
C. striatus	11	375	2%	84	30	14	16	11
C. striatus	11	450	2%	39	16	10	6	6
C. striatus	12	75	2%	79	30	15	15	0
C. striatus	12	150	2%	22	9	3	6	3
C. striatus	12	225	2%	62	16	11	5	3
C. striatus	12	300	2%	143	58	39	19	3
C. striatus	12	375	2%	80	45	19	26	5
C. striatus	12	450	2%	37	18	10	8	1
C. striatus	1	75	14%	412	81	72	9	1
C. striatus	1	150	14%	122	43	32	11	2
C. striatus	1	225	14%	162	60	42	18	1
C. striatus	1	300	14%	114	47	27	20	2
C. striatus	1	375	14%	27	17	7	10	2
C. striatus	1	450	14%	181	62	43	19	2
C. striatus	2	75	14%	203	43	38	5	0
C. striatus	2	150	14%	37	18	10	8	0
C. striatus	2	225	14%	36	18	10	8	0
C. striatus	2	300	14%	12	6	2	4	1

C. striatus	2	375	14%	4	4	0	4	2
C. striatus	2	450	14%	82	32	19	13	8
C. striatus	3	75	14%	116	26	22	4	0
C. striatus	3	150	14%	71	35	21	14	0
C. striatus	3	225	14%	62	37	18	19	0
C. striatus	3	300	14%	40	15	8	7	0
C. striatus	3	375	14%	15	13	2	11	0
C. striatus	3	450	14%	4	4	0	4	0
C. striatus	4	75	14%	65	25	16	9	0
C. striatus	4	150	14%	120	32	22	10	0
C. striatus	4	225	14%	15	10	2	8	5
C. striatus	4	300	14%	61	27	16	11	0
C. striatus	4	375	14%	12	8	4	4	1
C. striatus	4	450	14%	18	14	4	10	7
C. striatus	5	75	14%	103	33	26	7	3
C. striatus	5	150	14%	104	31	23	8	1
C. striatus	5	225	14%	67	29	19	10	4
C. striatus	5	300	14%	25	17	7	10	1
C. striatus	5	375	14%	45	23	12	11	1
C. striatus	5	450	14%	65	25	15	10	4
C. striatus	6	75	14%	165	40	34	6	1
C. striatus	6	150	14%	187	23	22	1	0
C. striatus	6	225	14%	140	24	20	4	1
C. striatus	6	300	14%	21	9	6	3	1
C. striatus	6	375	14%	95	24	17	7	3
C. striatus	6	450	14%	111	17	14	3	0
C. striatus	7	75	14%	51	27	9	18	5
C. striatus	7	150	14%	349	62	53	9	0
C. striatus	7	225	14%	137	33	23	10	3
C. striatus	7	300	14%	108	31	27	4	1
C. striatus	7	375	14%	42	18	11	7	2
C. striatus	7	450	14%	14	13	1	12	11
C. striatus	8	75	14%	41	13	10	3	0
C. striatus	8	150	14%	86	26	21	5	0
C. striatus	8	225	14%	7	6	1	5	1
C. striatus	8	300	14%	190	43	40	3	1
C. striatus	8	375	14%	126	28	23	5	2
C. striatus	8	450	14%	117	25	21	4	0
C. striatus	9	75	14%	64	21	13	8	0
C. striatus	9	150	14%	131	30	25	5	0
C. striatus	9	225	14%	22	11	7	4	1
C. striatus	9	300	14%	36	18	8	10	0
C. striatus	9	375	14%	21	14	5	9	0
C. striatus	9	450	14%	65	28	16	12	2

C. striatus	10	75	14%	112	30	24	6	1
C. striatus	10	150	14%	79	31	21	10	4
C. striatus	10	225	14%	144	40	30	10	3
C. striatus	10	300	14%	65	27	17	10	4
C. striatus	10	375	14%	25	12	7	5	1
C. striatus	10	450	14%	10	10	0	10	0
C. striatus	11	75	14%	151	27	25	2	1
C. striatus	11	150	14%	116	22	21	1	1
C. striatus	11	225	14%	72	23	19	4	3
C. striatus	11	300	14%	26	20	6	14	9
C. striatus	11	375	14%	10	6	3	3	3
C. striatus	11	450	14%	18	14	3	11	5
C. striatus	12	75	14%	155	39	29	10	0
C. striatus	12	150	14%	136	48	34	14	1
C. striatus	12	225	14%	120	36	30	6	3
C. striatus	12	300	14%	41	24	12	12	1
C. striatus	12	375	14%	62	39	20	19	5
C. striatus	12	450	14%	14	6	2	4	1
A. nigrofuscus	1	75	2%	20	5	4	1	0
A. nigrofuscus	1	150	2%	327	43	40	3	0
A. nigrofuscus	1	225	2%	75	10	8	2	0
A. nigrofuscus	1	300	2%	599	52	50	2	0
A. nigrofuscus	1	375	2%	629	55	51	4	0
A. nigrofuscus	1	450	2%	422	35	33	2	0
A. nigrofuscus	2	75	2%	217	40	33	7	0
A. nigrofuscus	2	150	2%	602	88	82	6	0
A. nigrofuscus	2	225	2%	162	37	25	12	0
A. nigrofuscus	2	300	2%	301	68	57	11	0
A. nigrofuscus	2	375	2%	203	59	43	16	0
A. nigrofuscus	2	450	2%	575	93	87	6	0
A. nigrofuscus	3	75	2%	287	51	49	2	0
A. nigrofuscus	3	150	2%	203	38	28	10	0
A. nigrofuscus	3	225	2%	627	68	67	1	0
A. nigrofuscus	3	300	2%	457	68	62	6	0
A. nigrofuscus	3	375	2%	333	56	51	5	0
A. nigrofuscus	3	450	2%	533	64	58	6	0
A. nigrofuscus	4	75	2%	95	17	17	0	0
A. nigrofuscus	4	150	2%	431	55	51	4	0
A. nigrofuscus	4	225	2%	4	4	0	4	0
A. nigrofuscus	4	300	2%	468	46	43	3	0
A. nigrofuscus	4	375	2%	46	13	8	5	0
A. nigrofuscus	4	450	2%	70	8	8	0	0
A. nigrofuscus	5	75	2%	790	96	88	8	0
A. nigrofuscus	5	150	2%	269	42	38	4	0

A. nigrofuscus	5	225	2%	472	89	78	11	0
A. nigrofuscus	5	300	2%	408	50	47	3	0
A. nigrofuscus	5	375	2%	421	68	61	7	0
A. nigrofuscus	5	450	2%	220	47	39	8	0
A. nigrofuscus	6	75	2%	269	35	31	4	0
A. nigrofuscus	6	150	2%	392	51	45	6	0
A. nigrofuscus	6	225	2%	190	39	34	5	0
A. nigrofuscus	6	300	2%	92	16	15	1	0
A. nigrofuscus	6	375	2%	363	46	41	5	0
A. nigrofuscus	6	450	2%	84	23	17	6	0
A. nigrofuscus	7	75	2%	184	28	26	2	0
A. nigrofuscus	7	150	2%	540	35	32	3	0
A. nigrofuscus	7	225	2%	413	47	42	5	0
A. nigrofuscus	7	300	2%	269	23	20	3	0
A. nigrofuscus	7	375	2%	272	26	25	1	0
A. nigrofuscus	7	450	2%	494	39	37	2	0
A. nigrofuscus	8	75	2%	213	42	37	5	0
A. nigrofuscus	8	150	2%	345	55	44	11	0
A. nigrofuscus	8	225	2%	482	72	62	10	0
A. nigrofuscus	8	300	2%	293	56	51	5	0
A. nigrofuscus	8	375	2%	524	78	72	6	0
A. nigrofuscus	8	450	2%	163	29	25	4	0
A. nigrofuscus	9	75	2%	202	50	37	13	0
A. nigrofuscus	9	150	2%	663	79	74	5	0
A. nigrofuscus	9	225	2%	95	21	20	1	0
A. nigrofuscus	9	300	2%	267	57	50	7	0
A. nigrofuscus	9	375	2%	33	11	10	1	0
A. nigrofuscus	9	450	2%	465	84	75	9	0
A. nigrofuscus	10	75	2%	573	94	84	10	0
A. nigrofuscus	10	150	2%	208	33	31	2	0
A. nigrofuscus	10	225	2%	289	56	48	8	0
A. nigrofuscus	10	300	2%	708	94	90	4	0
A. nigrofuscus	10	375	2%	498	68	63	5	0
A. nigrofuscus	10	450	2%	192	38	33	5	0
A. nigrofuscus	11	75	2%	551	56	55	1	0
A. nigrofuscus	11	150	2%	249	53	48	5	0
A. nigrofuscus	11	225	2%	324	50	44	6	0
A. nigrofuscus	11	300	2%	101	24	20	4	0
A. nigrofuscus	11	375	2%	117	23	18	5	0
A. nigrofuscus	11	450	2%	238	39	35	4	0
A. nigrofuscus	12	75	2%	75	28	14	14	0
A. nigrofuscus	12	150	2%	148	39	25	14	0
A. nigrofuscus	12	225	2%	99	29	19	10	0
A. nigrofuscus	12	300	2%	45	22	9	13	0

A. nigrofuscus	12	375	2%	83	29	19	10	0
A. nigrofuscus	12	450	2%	223	56	46	10	0
A. nigrofuscus	13	75	2%	29	14	10	4	0
A. nigrofuscus	13	150	2%	288	45	41	4	0
A. nigrofuscus	13	225	2%	324	50	44	6	0
A. nigrofuscus	13	300	2%	201	37	32	5	0
A. nigrofuscus	13	375	2%	252	42	34	8	0
A. nigrofuscus	13	450	2%	317	46	35	11	0
A. nigrofuscus	14	75	2%	262	68	48	20	0
A. nigrofuscus	14	150	2%	312	90	69	21	0
A. nigrofuscus	14	225	2%	97	28	19	9	0
A. nigrofuscus	14	300	2%	67	22	16	6	0
A. nigrofuscus	14	375	2%	295	80	65	15	0
A. nigrofuscus	14	450	2%	160	40	30	10	0
A. nigrofuscus	1	75	14%	542	49	46	3	0
A. nigrofuscus	1	150	14%	87	17	15	2	0
A. nigrofuscus	1	225	14%	389	56	51	5	0
A. nigrofuscus	1	300	14%	74	22	15	7	0
A. nigrofuscus	1	375	14%	115	22	17	5	0
A. nigrofuscus	1	450	14%	330	46	41	5	0
A. nigrofuscus	2	75	14%	173	48	36	12	0
A. nigrofuscus	2	150	14%	265	56	44	12	0
A. nigrofuscus	2	225	14%	487	75	71	4	0
A. nigrofuscus	2	300	14%	401	72	69	3	0
A. nigrofuscus	2	375	14%	377	88	75	13	0
A. nigrofuscus	2	450	14%	156	37	32	5	0
A. nigrofuscus	3	75	14%	427	54	52	2	0
A. nigrofuscus	3	150	14%	574	80	76	4	0
A. nigrofuscus	3	225	14%	162	45	37	8	0
A. nigrofuscus	3	300	14%	336	52	47	5	0
A. nigrofuscus	3	375	14%	458	59	57	2	0
A. nigrofuscus	3	450	14%	176	31	24	7	0
A. nigrofuscus	4	75	14%	396	60	52	8	0
A. nigrofuscus	4	150	14%	170	26	25	1	0
A. nigrofuscus	4	225	14%	502	54	48	6	0
A. nigrofuscus	4	300	14%	129	29	21	8	0
A. nigrofuscus	4	375	14%	9	4	3	1	0
A. nigrofuscus	4	450	14%	448	53	48	5	0
A. nigrofuscus	5	75	14%	424	79	67	12	0
A. nigrofuscus	5	150	14%	194	27	24	3	0
A. nigrofuscus	5	225	14%	673	93	89	4	0
A. nigrofuscus	5	300	14%	488	67	63	4	0
A. nigrofuscus	5	375	14%	622	89	84	5	0
A. nigrofuscus	5	450	14%	655	100	93	7	0

A. nigrofuscus	6	75	14%	422	60	55	5	0
A. nigrofuscus	6	150	14%	344	60	55	5	0
A. nigrofuscus	6	225	14%	352	61	54	7	0
A. nigrofuscus	6	300	14%	86	17	15	2	0
A. nigrofuscus	6	375	14%	175	39	32	7	0
A. nigrofuscus	6	450	14%	537	65	62	3	0
A. nigrofuscus	7	75	14%	54	7	6	1	0
A. nigrofuscus	7	150	14%	775	50	50	0	0
A. nigrofuscus	7	225	14%	110	14	13	1	0
A. nigrofuscus	7	300	14%	304	34	29	5	0
A. nigrofuscus	7	375	14%	28	7	4	3	0
A. nigrofuscus	7	450	14%	73	14	13	1	0
A. nigrofuscus	8	75	14%	182	32	29	3	0
A. nigrofuscus	8	150	14%	304	51	49	2	0
A. nigrofuscus	8	225	14%	315	54	46	8	0
A. nigrofuscus	8	300	14%	418	62	59	3	0
A. nigrofuscus	8	375	14%	488	52	48	4	0
A. nigrofuscus	8	450	14%	444	66	61	5	0
A. nigrofuscus	9	75	14%	234	45	39	6	0
A. nigrofuscus	9	150	14%	265	51	41	10	0
A. nigrofuscus	9	225	14%	21	8	5	3	0
A. nigrofuscus	9	300	14%	58	17	11	6	0
A. nigrofuscus	9	375	14%	420	82	73	9	0
A. nigrofuscus	9	450	14%	287	58	51	7	0
A. nigrofuscus	10	75	14%	556	70	64	6	0
A. nigrofuscus	10	150	14%	233	48	41	7	0
A. nigrofuscus	10	225	14%	384	68	62	6	0
A. nigrofuscus	10	300	14%	622	109	96	13	0
A. nigrofuscus	10	375	14%	238	62	50	12	0
A. nigrofuscus	10	450	14%	283	39	37	2	0
A. nigrofuscus	11	75	14%	382	49	48	1	0
A. nigrofuscus	11	150	14%	459	55	55	0	0
A. nigrofuscus	11	225	14%	224	34	33	1	0
A. nigrofuscus	11	300	14%	270	53	46	7	0
A. nigrofuscus	11	375	14%	59	20	16	4	0
A. nigrofuscus	11	450	14%	518	83	72	11	0
A. nigrofuscus	12	75	14%	321	47	43	4	0
A. nigrofuscus	12	150	14%	211	47	36	11	0
A. nigrofuscus	12	225	14%	110	29	23	6	0
A. nigrofuscus	12	300	14%	358	53	49	4	0
A. nigrofuscus	12	375	14%	136	29	25	4	0
A. nigrofuscus	12	450	14%	66	31	19	12	0
A. nigrofuscus	13	75	14%	357	38	37	1	0
A. nigrofuscus	13	150	14%	273	50	42	8	0

A. nigrofuscus	13	225	14%	420	56	51	5	0
A. nigrofuscus	13	300	14%	128	28	22	6	0
A. nigrofuscus	13	375	14%	160	39	26	13	0
A. nigrofuscus	13	450	14%	326	46	42	4	0
A. nigrofuscus	14	75	14%	346	71	61	10	0
A. nigrofuscus	14	150	14%	161	55	41	14	0
A. nigrofuscus	14	225	14%	236	62	48	14	0
A. nigrofuscus	14	300	14%	253	55	44	11	0
A. nigrofuscus	14	375	14%	249	74	57	17	0
A. nigrofuscus	14	450	14%	231	61	47	14	0
C. striatus	13	225	2%	60	26	20	6	0
C. striatus	13	225	6%	52	17	13	4	1
C. striatus	13	225	10%	13	11	2	9	1
C. striatus	13	225	14%	130	55	38	17	2
C. striatus	13	225	18%	124	43	29	14	0
C. striatus	13	225	22%	86	46	26	20	5
C. striatus	14	225	2%	40	14	8	6	0
C. striatus	14	225	6%	58	19	12	7	4
C. striatus	14	225	10%	139	58	34	24	4
C. striatus	14	225	14%	89	34	27	7	3
C. striatus	14	225	18%	102	46	24	22	1
C. striatus	14	225	22%	23	15	6	9	5
C. striatus	15	225	2%	59	23	12	11	1
C. striatus	15	225	6%	107	32	23	9	0
C. striatus	15	225	10%	47	19	14	5	0
C. striatus	15	225	14%	13	10	3	7	3
C. striatus	15	225	18%	108	40	29	11	0
C. striatus	15	225	22%	56	26	14	12	2
C. striatus	16	225	2%	110	24	21	3	2
C. striatus	16	225	6%	189	41	29	12	7
C. striatus	16	225	10%	32	19	5	14	0
C. striatus	16	225	14%	38	21	8	13	4
C. striatus	16	225	18%	65	32	19	13	1
C. striatus	16	225	22%	73	26	16	10	3
C. striatus	17	225	2%	96	24	19	5	8
C. striatus	17	225	6%	166	41	32	9	6
C. striatus	17	225	10%	160	45	35	10	4
C. striatus	17	225	14%	59	23	13	10	3
C. striatus	17	225	18%	74	32	20	12	4
C. striatus	17	225	22%	121	32	25	7	3
C. striatus	18	225	2%	49	12	8	4	1
C. striatus	18	225	6%	33	12	7	5	2
C. striatus	18	225	10%	13	11	1	10	2
C. striatus	18	225	14%	89	30	23	7	1

C. striatus	18	225	18%	40	16	10	6	1
C. striatus	18	225	22%	45	17	11	6	4
C. striatus	19	225	2%	76	31	16	15	2
C. striatus	19	225	6%	125	48	28	20	2
C. striatus	19	225	10%	55	20	13	7	1
C. striatus	19	225	14%	76	34	21	13	1
C. striatus	19	225	18%	44	17	11	6	4
C. striatus	19	225	22%	19	10	7	3	2
C. striatus	20	225	2%	16	7	4	3	0
C. striatus	20	225	6%	48	22	11	11	1
C. striatus	20	225	10%	68	21	15	6	0
C. striatus	20	225	14%	102	29	24	5	2
C. striatus	20	225	18%	49	23	11	12	2
C. striatus	20	225	22%	70	31	15	16	1
