# Supplementary material for: The Effects of Algal Turf Sediments and Organic Loads on Feeding by Coral Reef Surgeonfishes
Source: PLoS One. 2017 Jan 3;12(1):e0169479. doi: 10.1371/journal.pone.0169479 (PMC5207718; doi:10.1371/journal.pone.0169479)
Supplement: S2 Table — SE = standard error. Negative binomial models used a t statistic while binomial models used a z statistic. (PDF) [file pone.0169479.s002.pdf]

S2 Table. Summary of GLMM results used to examine the effects of sediment and organic loads on *Ctenochaetus striatus*. SE = standard error. Negative binomial models used a *t* statistic while binomial models used a *z* statistic.

**Response variable**	**Model used**	**Predictor variable**	**Estimate**	**SE**	***t/z* value**	***p* value**
**Sediment Load Experiment**						
Number of Bites	Negative-binomial (GLMM)	Intercept<br>Sediment Load	5.0339<br>-0.0027	0.1334<br>0.0004	37.72<br>-6.35	<0.0001<br><0.0001
Proportion of bites rejected	Binomial (GLMM)	Intercept<br>Sediment Load	-5.7662<br>0.0070	0.3472<br>0.0010	-16.61<br>7.27	<0.0001<br><0.0001
Proportion of feeding bouts with multiple bites	Binomial (GLMM)	Intercept<br>Sediment Load	1.4865<br>-0.0035	0.1540<br>0.0005	9.654<br>-6.915	<0.0001<br><0.0001
**Organic Load Experiment**						
Number of Bites	Negative-binomial (GLMM)	Intercept<br>Organic Load	4.3616<br>-0.0064	0.1819<br>0.0129	23.976<br>-0.499	<0.0001<br>0.618
Proportion of bites rejected	Binomial (GLMM)	Intercept<br>Organic Load	-3.995<br>0.0348	0.3237<br>0.0220	-12.342<br>1.583	<0.0001<br>0.113
Proportion of feeding bouts with multiple bites	Binomial (GLMM)	Intercept<br>Organic Load	0.6937<br>-0.0143	0.1881<br>0.0134	3.689<br>-1.067	<0.001<br>0.286
