# Supplementary material for: The Effects of Algal Turf Sediments and Organic Loads on Feeding by Coral Reef Surgeonfishes
Source: PLoS One. 2017 Jan 3;12(1):e0169479. doi: 10.1371/journal.pone.0169479 (PMC5207718; doi:10.1371/journal.pone.0169479)
Supplement: S3 Table — Models are compared using the corrected Akaike Information Criterion (AICc). Shown are degrees of freedom (df), model maximum log-likelihood (logLik), AICc, change in AICc (Δ) and AICc weight (wAICc). (PDF) [file pone.0169479.s003.pdf]

S3 Table. Comparison of GLMMs used to examine the response of *Ctenochaetus striatus* to sediment and organic loads. Models are compared using the corrected Akaike Information Criterion (AICc). Shown are degrees of freedom (df), model maximum log-likelihood (logLik), AICc, change in AICc (Δ) and AICc weight (wAICc).

Model	Variables	df	logLik	AICc	Δ	wAICc
Bite count response to sediment loads	Sediment	4	-749.71	1507.71	0.00	0.66
	Sediment + Organics	5	-749.71	1509.86	2.15	0.23
	Sediment × Organics	6	-749.30	1511.22	3.51	0.11
	Null	3	-767.34	1540.86	33.15	0.00
	Organics	4	-767.27	1542.84	35.13	0.00
Proportion of bites rejected	Sediment	4	-289.97	588.23	0.00	0.54
	Sediment + Organics	5	-289.41	589.26	1.03	0.32
	Sediment × Organics	6	-289.12	590.85	2.62	0.14
	Null	3	-313.03	632.23	43.99	0.00
	Organics	4	-312.53	633.35	45.12	0.00
Proportion of feeding bouts with multiple bites	Sediment	4	-395.66	799.60	0.00	0.60
	Sediment + Organics	5	-395.50	801.44	1.84	0.24
	Sediment × Organics	6	-394.82	802.25	2.65	0.16
	Null	3	-416.53	839.23	39.63	0.00
	Organics	4	-416.42	841.13	41.53	0.00
Bite count response to organic loads	Null	3	-243.3	493.14	0.00	0.74
	Organics	4	-243.17	495.28	2.14	0.26
Proportion of bites rejected	Organics	4	-95.35	199.63	0.00	0.52
	Null	3	-96.63	199.81	0.18	0.48
Proportion of feeding bouts with multiple bites	Null	3	-129.32	265.19	0.00	0.62
	Organics	4	-128.76	266.45	1.27	0.35
