# Supplementary material for: The Effects of Algal Turf Sediments and Organic Loads on Feeding by Coral Reef Surgeonfishes
Source: PLoS One. 2017 Jan 3;12(1):e0169479. doi: 10.1371/journal.pone.0169479 (PMC5207718; doi:10.1371/journal.pone.0169479)
Supplement: S4 Table — SE = standard error. Negative binomial models used a t statistic while binomial models used a z statistic. (PDF) [file pone.0169479.s004.pdf]

S4 Table. Summary of GLMM results used to examine the effects of sediment and organic loads on *Acanthurus nigrofuscus*. SE = standard error. Negative binomial models used a *t* statistic while binomial models used a *z* statistic.

**Response variable**	**Model used**	**Predictor variable**	**Estimate**	**SE**	***t/z* value**	***p* value**
Number of Bites	Negative-binomial (GLMM)	Intercept<br>Sediment Load	5.7371<br>-0.0002	0.1264<br>0.0004	45.40<br>-0.55	<0.0001<br>0.582
Proportion of feeding bouts with multiple bites	Binomial (GLMM)	Intercept<br>Organic Load	2.0591<br>-0.1499	0.1247<br>0.1042	16.518<br>-1.438	<0.0001<br>0.151
