# Supplementary material for: The Effects of Algal Turf Sediments and Organic Loads on Feeding by Coral Reef Surgeonfishes
Source: PLoS One. 2017 Jan 3;12(1):e0169479. doi: 10.1371/journal.pone.0169479 (PMC5207718; doi:10.1371/journal.pone.0169479)
Supplement: S5 Table — Models are compared using the corrected Akaike Information Criterion (AICc). Shown are degrees of freedom (df), model maximum log-likelihood (logLik), AICc, change in AICc (Δ) and AICc weight (wAICc). (PDF) [file pone.0169479.s005.pdf]

S5 Table. Comparison of GLMMs used to examine the response of *Acanthurus nigrofuscus* to sediment and organic loads. Models are compared using the corrected Akaike Information Criterion (AICc). Shown are degrees of freedom (df), model maximum log-likelihood (logLik), AICc, change in AICc (Δ) and AICc weight (wAICc).

**Model**	**Variables**	**df**	**logLik**	**AICc**	**Δ**	**wAICc**
Bite count response to sediment loads	Null	3	-1103.42	2212.99	0.00	0.50
	Sediment	4	-1103.27	2214.79	1.80	0.21
	Organics	4	-1103.37	2214.99	1.99	0.19
	Sediment + Organics	5	-1103.22	2216.82	3.82	0.07
	Sediment × Organics	6	-1103.06	2218.65	5.65	0.03
Proportion of feeding bouts with multiple bites	Null	3	-440.84	887.83	0.00	0.27
	Organic	4	-439.83	887.90	0.08	0.26
	Sediment	4	-440.27	888.78	0.5	0.17
	Sediment + Organics	5	-439.25	888.87	1.04	0.16
	Sediment × Organics	6	-438.28	889.08	1.25	0.14
